# A novel enzyme-linked immunosorbent assay for detection of *Escherichia coli* O157:H7 using immunomagnetic and beacon gold nanoparticles

**DOI:** 10.1186/1757-4749-6-14

**Published:** 2014-05-20

**Authors:** Zhiqiang Shen, Nannan Hou, Min Jin, Zhigang Qiu, Jingfeng Wang, Bin Zhang, Xinwei Wang, Jie Wang, Dongsheng Zhou, Junwen Li

**Affiliations:** 1 Tianjin Institute of Health and Environmental Medicine, Key Laboratory of Risk Assessment and Control for Environment and Food Safety, Tianjin 300050, China; 2 State Key Laboratory of Pathogen and Biosecurity, Beijing Institute of Microbiology and Epidemiology, Beijing 100071, China

**Keywords:** *Escherichia coli* O157:H7, ELISA, Immunomagnetic nanoparticles, Beacon gold nanoparticles

## Abstract

This paper presents a functional nanoparticle-enhanced enzyme-linked immunosorbent assay (FNP-ELISA) for detection of enterohemorrhagic *Escherichia coli* (EHEC) O157:H7. Immunomagnetic nanoparticles (IMMPs) conjugated with monoclonal anti-O157:H7 antibody were used to capture *E. coli* O157:H7. Beacon gold nanoparticles (B-GNPs) coated with polyclonal anti-O157:H7 and biotin single-stranded DNA (B-DNA) were then subjective to immunoreaction with *E. coli* O157:H7, which was followed by streptavidin-horseradish peroxidase (Strep-HRP) conjugated with B-GNPs based on a biotin-avidin system. The solutions containing *E. coli* O157:H7, IMMPs, B-GNPs, and Strep-HRP were collected for detecting color change. The signal was significantly amplified with detection limits of 68 CFU mL^-1^ in PBS and 6.8 × 10^2^ to 6.8 × 10^3^ CFU mL^-1^ in the food samples. The FNP-ELISA method developed in this study was two orders of magnitude more sensitive than immunomagnetic separation ELISA (IMS-ELISA) and four orders of magnitude more sensitive than C-ELISA. The entire detection process of *E. coli* O157:H7 lasted only 3 h, and thus FNP-ELISA is considered as a time-saving method.

## Introduction

The World Health Organization estimated that about 1.8 million people worldwide die every year from diarrheal diseases, which are often caused by consuming microbiologically contaminated food or by drinking water [1]. Among the pathogens causing diarrheal diseases, enterohemorrhagic *Escherichia coli* (EHEC) strains are prominently responsible for serious foodborne outbreaks [2,3]. In particular, *E. coli* O157:H7, a predominant strain of EHEC that was first isolated and recognized as a new type of intestinal pathogenic bacterium in the United States in 1982 [4], has become a global public health problem. *E. coli* O157:H7 outbreaks have occurred in many developing and developed countries, causing huge health care costs and product recalls. The Center for Disease Control and Prevention of the United States estimated that 73,000 cases of illness and 61 deaths per year in the United States are caused by *E. coli* O157:H7 [5].

The development of a rapid and reliable detection of *E. coli* O157:H7 has become highly important for food safety and public health [6]. However, traditional methods for the detection of *E. coli* O157:H7 encompassing enrichment, plating, culturing, enumeration, biochemical testing, and microscopic examination can take up to 60 h, thereby being laborious and time-consuming [7]. Polymerase chain reactions (PCRs), including simple PCR [8], multiplex PCR [9,10], and real-time PCR [11,12], are commonly used for rapid detection of *E. coli* O157:H7, but require complex set-ups and well-trained personnel. In addition, some very sensitive and selective but expensive, complicated, and time-consuming methods have been applied in the detection of *E. coli* O157:H7, especially including immunomagnetic separation (IMS) analysis [13], flow cytometry [14], fluorescence in situ hybridization [15], DNA microarrays [16], and several label-free methods (such as surface plasmon resonance [17] and use of electrochemical impedance immunosensors [18,19]).

Enzyme-linked immunosorbent assay (ELISA) was reported to quantitatively detect immunoglobulin G in 1971 [20]. Conventional ELISA (C-ELISA) has high reproducibility and possibility for the simultaneous quantification of a great number of assays, and is widely used to detect the presence of substances, including bacteria [21], viruses [22], proteins [23], and pesticides [24]. However, the detection limit of C-ELISA to *E. coli* O157:H7 is only 10^5^ to 10^7^ CFU mL^-1^[25], which is inadequate when the infectious dose is lower than 100 cells [26].

In recent years, the emergence of nanotechnology is opening new horizons for high detection limits in biological fields [27-30]. Nanoparticles of various shapes, sizes, and compositions have broad applications in microorganism detection [31,32]. Much attention has been focused on amplifying the detection signal using nanoparticles [33,34], which can enhance enzyme activity [35,36]. Magnetic and gold particles have been used to improve the detection limit of ELISA [30,37].

In this study, we developed a functional nanoparticle-enhanced ELISA (FNP-ELISA) using immunomagnetic nanoparticles (IMMPs) and beacon gold nanoparticles (B-GNPs) for detecting *E. coli* O157:H7. The detection limit of *E. coli* O157:H7 by the developed FNP-ELISA is much higher than that of C-ELISA or immunomagnetic separation ELISA (IMS-ELISA), and thus FNP-ELISA had the highest sensitivity compared to the other ELISA methods.

## Materials and methods

### Reagents and materials

Rabbit polyclonal anti-*E. coli* O157:H7 antibody and mouse monoclonal anti-O157:H7 antibody were prepared and purified in our laboratory. Single-stranded DNA 5′(biotin)-GCTAGTGAACACAGTT-GTGTAAAAAAAAAA (SH)-3′ was synthesized by Sangon Biotech Co., Ltd. (China). Streptavidin-horseradish peroxidase (Strep-HRP) and peroxidase-conjugated affinipure goat anti-rabbit IgG (IgG-HRP) were purchased from Beijing Biosynthesis Biological Technology Co., Ltd. (China). Bovine serum albumin (BSA), 3,3′,5,5′- tetramethylbenzidine (TMB-H_2_O_2_), and hydrogen tetrachloroaurate (III) trihydrate (HAuCl_4_ · 3H_2_O, 99.9%) were purchased from Sigma-Aldrich (USA). Dextran with a molecular weight of 40,000 (T-40) was obtained from Pharmacia (GE Healthcare, USA). Sorbitol-MacConkey agar (SMAC) and xylose-lysine-tergitol 4 (XLT4) agar were purchased from Difco (Becton Dickinson, USA). Ferric chloride hexahydrate (FeCl_3_ · 6H_2_O), ferrous chloride tetrahydrate (FeCl_2_ · 4H_2_O), and other chemicals were of analytically pure grade or better quality. The buffer solutions were prepared in our laboratory. All aqueous solutions were prepared using ultrapure water (18.0 MΩ/cm) as required.

### Preparation of microbial samples

*E. coli* O157:H7 strain 35150 and *E. coli* K12 were obtained from the American Type Culture Collection (ATCC, USA). *Salmonella senftenberg* 50315, *Shigella sonnei* 51081, and *E. coli* O157:Hund strain 21531 (Hund indicated that H antigen was not determined) [38] were obtained from the Institute of Epidemiology and Microbiology, Academy of Preventive Medical Sciences of China. Pure cultures of bacteria were grown in nutrient broth at 37°C for 24 h before use. The concentrations of *E. coli* O157:H7, O157:Hund, and K12 were determined by the conventional surface plate count method using SMAC. *S. senftenberg* and *S. sonnei* were enumerated using XLT4 agar. The cultured bacteria were divided into two portions. The first portion was placed in a boiling water bath for 20 min to kill the bacterial cells, and diluted to the desired concentration with PBS (0.01 M, pH 7.4) for ELISA detection. The second portion was not heated because the number of living cells was counted.

Milk, vegetable, and ground beef were purchased from a local market in Tianjin (China), and each weighed 25 g (mL) for detection. The killed *E. coli* O157:H7 solution was transferred into a small vial equipped with an atomizer. The mists of *E. coli* O157:H7 inoculums were sprayed onto the three samples, and the samples were then stored at 4 ± 1°C for 1 h. Each sample was added to 0.25 mL of *E. coli* O157:H7 solution. The samples were placed into sterile filter stomacher bags, and macerated in 225 mL of PBS with a stomacher blender (Bilon-8 Bilang Co. Ltd., Beijing, China) at 200 rpm for 2 min. The homogenate was serially diluted in PBS for ELISA detection. The negative samples that were not added to *E. coli* O157:H7 solution were analyzed according to the Chinese National Standard Method GB/T 4789.36-2008 [39].

### Preparation of IMMPs

Magnetic nanoparticles (MPs) were prepared from FeCl_3_, FeCl_2_, ammonia solution, and dextran (T-40), and oxidized with NaIO_4_ as described previously [40]. Mouse monoclonal anti-*E. coli* O157:H7 antibody (0.5 mg/mL) was added to the oxidized MP suspension at a ratio of 0.3:1, mixed thoroughly, and incubated in the dark at 4°C for approximately 24 h. IMMPs were washed three times with PBS by placing a magnetic plate against the side wall of the tubes for 5 min to concentrate the particles into the pellets on the side walls. The supernatant was discarded using a transferpettor. The pellets were resuspended in 1 mL of PBST (0.05% Tween-20 in 0.01 M PBS, pH 7.4). BSA was then added to a final concentration of 1% to block any unreacted or nonspecific site. The amount of IMMPs, incubation time, and separation time varied to determine their effects on the recovery of O157:H7 (details in Supplementary Materials).

### Preparation of GNPs

GNPs were prepared according to the literature with slight modifications [41]. In brief, 2 mL of 1% HAuCl_4_ was mixed with 198 mL of fresh ultrapure water. The mixture was stirred vigorously with a magnetic agitator while being heated in a boiling water bath for 20 min, followed by the rapid addition of 5 mL of 1% sodium citrate solution. After the color finally turned to full red, the mixture was stirred again for 10 min before cooling to room temperature. The GNP solution was filtered through a 0.22 μm cellulose nitrate filter to remove any floating aggregates. The prepared GNPs were characterized using a transmission electron microscope (TEM; Tecnai G2 F20, FEI, Netherlands) and ultraviolet spectrophotometer (UV 2500, Shimadzu, Japan).

### Preparation of various B-GNPs

B-GNPs were prepared following a previously reported procedure [42,43] with slight modifications. Rabbit polyclonal anti-*E. coli* O157:H7 antibody (7 μg) was added to 1 mL of pH-adjusted GNP solution (pH 8.2) and incubated at room temperature for 30 min. The mixture was added with 30 μL of different concentrations of B-DNA, and incubated in the dark at 4°C for more than 16 h. Approximately 100 μL of 1% sodium chloride solution was then added to the mixture, and incubated at 4°C for 3 h. BSA was added to a final concentration of 1% to block any unreacted or nonspecific site. The prepared B-GNP solution was centrifuged at 20,000 *g* for 1 h at 4°C. The final deposition was suspended in 0.5 mL of storage buffer (PBS, 0.01 M, pH 7.4, 1% BSA, 0.02% NaN_3_) and stored at 4°C.

### C-ELISA

Mouse monoclonal anti-*E. coli* O157:H7 antibody (100 μL of 5 mg L^-1^) was added to a 96-well plate and incubated at 37°C for 2 h. The plate was rinsed with PBST (0.05% Tween-20 in 0.01 M PBS, pH 7.4) three times to remove unbound antibodies, followed by the addition of 100 μL of PBS-BSA (1% BSA in 0.01 M PBS, pH 7.4) and incubation at 4°C for 12 h. Different concentrations of *E. coli* O157:H7 (100 μL) were added to each well and reacted at 37°C for 1 h. After rinsing three times, 100 μL of 5 mg L^-1^ rabbit polyclonal anti-*E. coli* O157:H7 antibody was added to the plate incubated at 37°C for 1 h. Subsequently, 100 μL of 0.02 mg L^-1^ IgG-HRP was added to the plate and incubated for 1 h at 37°C. The plate was rinsed three times to remove unbound IgG-HRP. Finally, 100 μL of TMB-H_2_O_2_ solution was added to each well and incubated at 37°C for 15 min. The reaction was terminated using 100 μL of 0.5 M sulfuric acid, and the absorbance at 450 nm was measured using a microplate reader.

### IMS-ELISA

*E. coli* O157:H7 was separated using IMMPs according to the previous procedure in Supplementary Materials, and the particle-bacteria complex (100 μL) was finally resuspended. Rabbit polyclonal anti-O157:H7 antibody (100 μL of 5 mg L^-1^) was added to the complex, and incubated at room temperature for 30 min. The unbound antibody was removed by the magnetic plate method. The particle-bacteria-antibody complex was resuspended using 100 μL of 0.02 mg L^-1^ IgG-HRP, and incubated for 1 h at 37°C. The complex was resuspended and transferred to a 96-well plate after excess IgG-HRP was removed by the magnetic plate method. Finally, TMB-H_2_O_2_ and sulfuric acid were subsequently added, and the plate was read at 450 nm using a microplate reader.

### FNP-ELISA

IMMPs (10 μL) were added to 1 mL of *E. coli* O157:H7 suspension at 10^6^ CFU mL^-1^ in a 1.5 mL Eppendorf tube. The tube was carefully inverted several times and incubated at room temperature for 10 min. Approximately 100 μL of the complex of *E. coli* O157:H7 and IMMPs was obtained by the magnetic plate method. Various B-GNPs (100 μL) were added to 100 μL of the complex, and incubated at room temperature for 30 min. The unbound B-GNPs were removed by the magnetic plate method, and the complex was rinsed three times with PBST. Subsequently, 100 μL of Strep-HRP (0.01 mg L^-1^) solution was added to the Eppendorf tube and incubated at 37°C for 1 h. The unbound Strep-HRP was removed by the magnetic plate method. The final complex was washed three times with PBST by the magnetic plate method, and resuspended in 100 μL of PBS. The suspension was then transferred to a 96-well plate. Finally, TMB-H_2_O_2_ and sulfuric acid were subsequently added, and the plate was read at 450 nm using a microplate reader.

### Experimental replicates and statistical methods

All the experiments were done with at least biological replicates, and the values were expressed as mean ± standard deviation. A conventionally used positive control to negative control (P/N) value ≥2.1 was considered positive in the three ELISA methods [44]. When needed, paired Student’s *t-*test was performed to determine statistically significant differences; *P* <0.01 was considered to indicate statistical significance.

## Results and discussion

### Properties of IMMS

We prepared MPs that were roughly spherical in shape with diameters ranging from 40–60 nm, and contained an electron-dense core of 5 nm (Additional file 1: Figure S1). We also prepared IMMPs, and the recovery of IMMPs increased with increasing amount of IMMPs and incubation time. Relatively high recovery was obtained with 10 μL of IMMPS (Additional file 1: Table S1) and 10 min of incubation (Additional file 1: Table S2). The optimal time for separation was 2 min (Additional file 1: Table S3). This finding was supported by a previous study, which showed that target cells can be separated from samples using MPs coupled with aptamer/nucleic acid/antibody [45,46].

### Properties of B-GNPs

**Figure 1:**
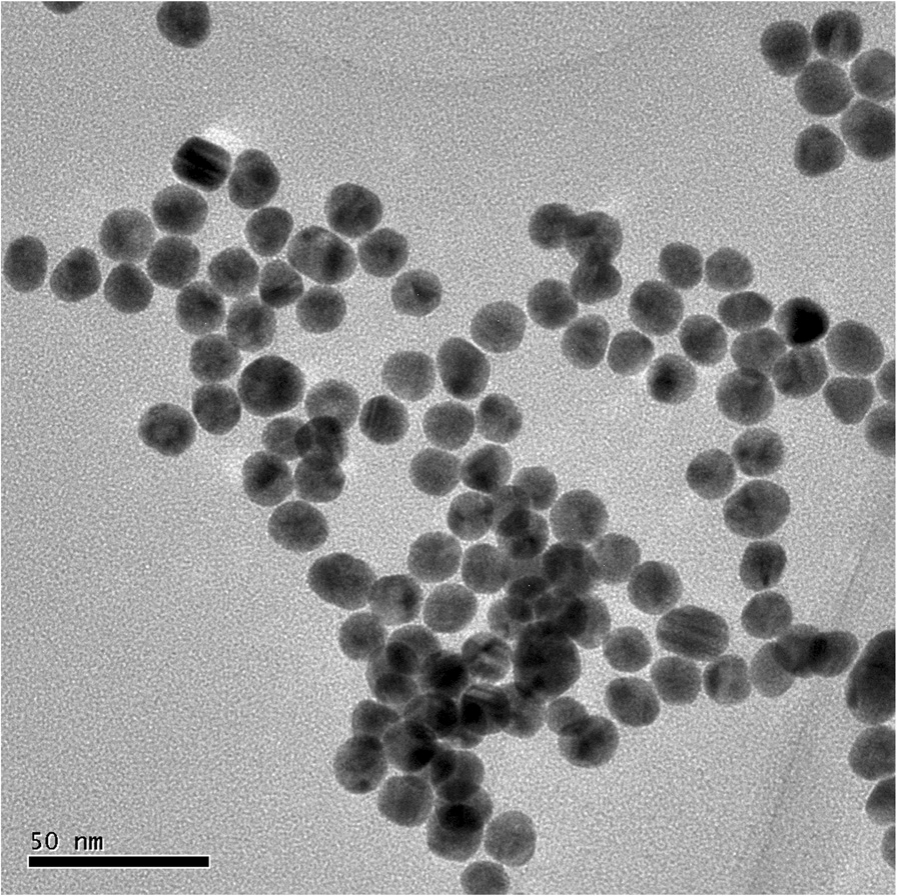
Typical TEM images of GNPs.

We successfully prepared GNPs with an average diameter of approximately 18 nm as measured by TEM (Figure 1). The maximum peak of GNP solution was 518 nm as determined by UV scanning. We prepared a new type of GNP, namely, B-GNPs, which could target *E. coli* O157:H7 through the polyclonal antibody and amplify signals through the biotin-avidin Strep-HRP system.

### Experimental design for FNP-ELISA

**Figure 2:**
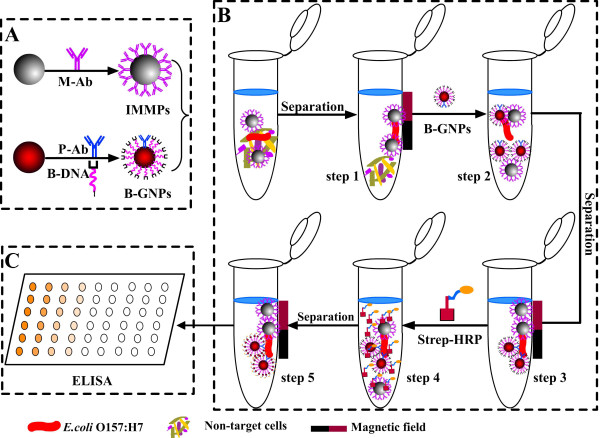
**Flow chart of FNP-ELISA.** Preparation of IMMPs and B-GNPs **(part A)**; **(part B)** comprises five steps, namely, magnetic separation of target cells (step 1), conjugation of B-GNPs (step 2), removal of free B-GNPs (step 3), conjugation of Strep-HRP (step 4), and removal of free Strep-HRP (step 5); and FNP-ELISA detection **(part C)**.

In this study, a novel ELISA method namely FNP-ELISA was established to detect *E. coli* O157:H7, in comparison with C-ELISA, and IMS-ELISA. A schematic diagram illustrating the detection of *E. coli* O157:H7 by FNP-ELISA is presented in Figure 2.

First, functional monoclonal anti-*E. coli* O157:H7-conjugated IMMPs and polyclonal anti-*E. coli* O157:H7 antibody and B-GNPs are prepared (A). Second, IMMPs are mixed with a sample to target *E. coli* O157:H7, and the O157:H7-IMMP complex is separated using the magnetic plate method (step 1, B). Third, B-GNPs are added to target *E. coli* O157:H7 in the *E. coli* O157:H7-IMMP complex (step 2, B), and the unbound B-GNPs are removed by the magnetic plate method (step 3, B). Fourth, Strep-HRP is added to react with polyclonal anti-*E. coli* O157:H7 (step 4, B), and unbound Strep-HRP is removed by the magnetic plate method. Finally, the remaining routine ELISA steps are completed (C).

## Optimized amounts of B-DNA, Strep-HRP and B-GNPs for FNP-ELISA

**Figure 3:**
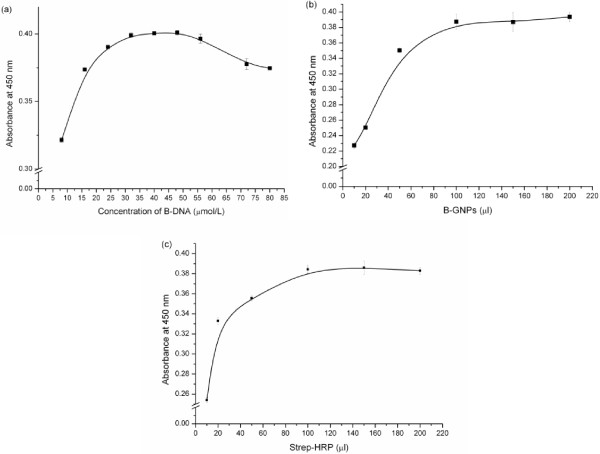
**Optimal amounts of B-DNA, Strep-HRP, and B-GNPs. (a)** Optimization graph of the B-DNA concentration in the preparation of B-GNPs. **(b)** Optimization of the B-GNP volume. **(c)** Strep-HRP volume optimization graph.

The optimal amounts of B-DNA, Strep-HRP, and B-GNPs for FNP-ELISA were investigated (Figure 3). To obtain an optimized concentration of B-DNA, we added 100 μL of different B-GNPs conjugated with different concentrations of B-DNA to the complex of *E. coli* O157:H7 and IMMPs after separating *E. coli* O157:H7. The aforementioned procedure was then repeated.

The optimal volume of B-GNPs was determined by adding different volumes of the optimized B-GNPs to the complex of *E. coli* O157:H7 and IMMPs after separating *E. coli* O157:H7. The detection procedure was then operated as above.

The optimized volume of Strep-HRP was determined when the optimized volume of B-GNPs was applied in the procedure. After optimizing the parameters, various concentrations of *E. coli* O157:H7 were detected in PBS using FNP-ELISA. The negative samples included K12, *S. senftenberg*, and *S. sonnei* in FNP-ELISA. This method was then used to determine the concentration of *E. coli* O157:H7 in the milk, vegetable, and ground beef samples.

We found that the FNP-ELISA signal increased with increasing B-DNA concentration (Figure 3a), and the optimal concentration of B-DNA was 40 μmol L^-1^. This finding was also observed in a previous study, which reported that excess B-DNA molecules cause excessive HRP molecules to bind with GNPs, posing steric hindrance to hamper antigens from access to antibodies on GNPs [47].

The signal of FNP-ELISA was strongly dependent on the amount of B-GNPs. The ELISA signal increased as the amount of B-GNPs increased, and reached a plateau at 100 μL (Figure 3b). Thus, we used 100 μL of B-GNPs in subsequent experiments. The optimal amount of Strep-HRP was also obtained at 100 μL (Figure 3c).

### Comparison of FNP-ELISA to C-ELISA and IMS-ELISA

Functional nanoparticles were used to improve the sensitivity of ELISA, and the results were compared simultaneously. The blank, positive, and negative controls were PBS, *E. coli* O157:H7 (10^6^ CFU mL^-1^), and *E. coli* O157:Hund (10^6^ CFU mL^-1^), respectively. These controls were included on each plate in the experiments.

**Figure 4:**
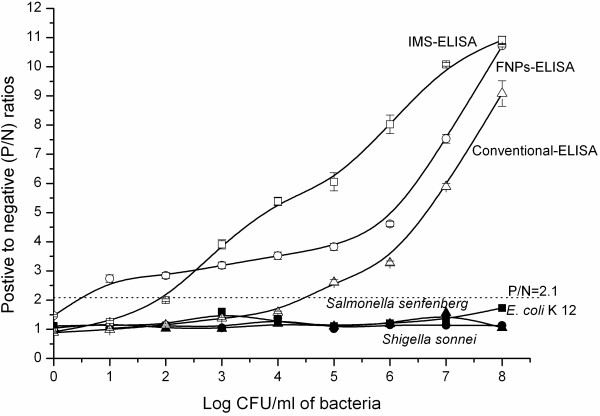
**ELISA Detection sensitivity.** The detection limits of C-ELISA, IMS-ELISA, and FNP-ELISA were 6.8 × 10^5^ (1.8 ≤ RSD ≤ 4.7), 6.8 × 10^3^ (0.6 ≤ RSD ≤ 5.2), and 6.8 × 10^1^ CFU mL^-1^ (0.3 ≤ RSD ≤ 4.7), respectively (*n* = 3).

The surface plate counts showed that the concentrations of the test solutions of *E. coli* O157:H7, *E. coli* O157: Hund, K12, *S. senftenberg*, and *S. sonnei* were 6.8 × 10°–6.8 × 10^8^ CFU mL^-1^, 6.1 × 10^6^ CFU mL^-1^, 7.1 × 10°–7.1 × 10^8^ CFU mL^-1^, 8.1 × 10°–8.1 × 10^8^ CFU mL^-1^, and 2.3 × 10°–2.3 × 10^8^ CFU mL^-1^, respectively. The detection limits of C-ELISA, IMS-ELISA, and FNP-ELISA were 6.8 × 10^5^ (*n* = 3, 1.8 ≤ RSD ≤ 4.7), 6.8 × 10^3^ (*n* = 3, 0.6 ≤ RSD ≤ 5.2), and 68 CFU mL^-1^ (*n* = 3, 0.3 ≤ RSD ≤ 4.7) in PBS, respectively (Figure 4). The three ELISA methods had high reproducibility, and all RSD values were lower than 10.

The specificity of the three ELISA methods was dependent on the quality of the antibody. The specificity of the rabbit polyclonal anti-*E. coli* O157:H7 antibody and mouse monoclonal anti-*E. coli* O157:H7 antibody was tested using 61 bacteria, and false negative and false positive results were not observed (data not shown). *E. coli* O157:H7 is a common intestinal pathogen, so *S. senftenberg* and *S. sonnei*, which are also common intestinal pathogens, were selected as negative samples. *E. coli* K12, which represented *E. coli*, was another negative sample. The P/N values of the negative signals were all significantly lower than 2.1 (Figure 4), which shows that FNP-ELISA had high specificity.

*E. coli* O157:H7 was not detected in the raw vegetable, milk, and ground beef samples using GB/T method 4789.36-2008. The detection limits of FNP-ELISA were 6.8 × 10^2^ CFU mL^-1^ in vegetable and milk, and 6.8 × 10^3^ CFU mL^-1^ in ground beef. The reduction in sensitivities may be attributed to the loss of some functional nanoparticles and targeting bacteria in the food residues, particularly grease foods.

### Concluding remarks

Multiple ultrasensitive methods for detecting *E. coli* O157:H7 have been reported [48,49], but they usually require expensive equipment or skilled personnel and thus have difficulty in wide use. C-ELISA has been widely established to detect microorganisms, proteins, pesticides, and heavy metals because of its simplicity and low cost, but its application is limited because of its low detection limit. Data presented here indicated that FNP-ELISA had a high sensitivity in detecting *E. coli* O157:H7, with a detection limit of 68 CFU mL^-1^ in PBS and 6.8 × 10^2^ to 10^3^ CFU mL^-1^ in foods. The detection limit of FNP-ELISA was about two or four orders of magnitude lower than that of IMS-ELISA or C-ELISA, respectively. Moreover, the total analysis time of FNP-ELISA was only approximately 3 h. Therefore, FNP-ELISA may be used for detection of *E. coli* O157:H7 in foods, and also for other microorganisms if appropriate antibodies are available.

## Competing interests

The authors declare that they have no competing interests.

## Authors’ contribution

DZ and JL conceived and designed the experiments. ZS, NH, MJ, ZQ, JW, BZ, XW and JW performed the experiments. ZS, DZ and JL analyzed the data. ZS, DZ and JL drafted the manuscript. All authors read and approved the final manuscript.

## Supplementary Material

Additional file 1: Table S1 Effect of amount of IMMPs on recovery. **Table S2** Effect of incubation time on recovery. **Table S3** Effect of separation on recovery. **Figure S1**  Transmission electron micrograph of IMMPs. The electron dense core was about 5 nm. Click here for file
