# Supplementary material for: A novel enzyme-linked immunosorbent assay for detection of Escherichia coli O157:H7 using immunomagnetic and beacon gold nanoparticles
Source: Gut Pathog. 2014 May 20;6:14. doi: 10.1186/1757-4749-6-14 (PMC4033681; doi:10.1186/1757-4749-6-14)
Supplement: Additional file 1: Table S1 — Effect of amount of IMMPs on recovery. Table S2 Effect of incubation time on recovery. Table S3 Effect of separation on recovery. Figure S1 Transmission electron micrograph of IMMPs. The electron dense core was about 5 nm. [file 1757-4749-6-14-S1.doc]

**Additional file 1**

**Determination of the volume of IMMPs, incubation time and separation time**

The *E. coli* O157:H7 suspension diluted to 10^2^ CFU ml^-1^ was used as the sample. Various volumes of IMMPs were added to 1 ml of sample in 1.5-ml Eppendorf tube which were gently inverted several times. The suspensions were incubated at room temperature on a rotary shaker (20 rpm) for 60 min. The particles were separated by placing a magnetic plate against the side wall of the tubes, and the suspension was left for 30 min to concentrate the particles into the pellets on the side of the tubes. The supernatant was discarded using a transferpettor. The magnetic plate was removed, and 1 ml washing buffer (0.05% Tween-20 in 0.01 M PBS, pH 7.4) was added. The separation procedure was repeated three times. Eventually, the particle-bacteria complex was resuspended in 100 μl of PBS, and the suspension was transferred onto medium selective for *E.coli* O157:H7 (SMAC). The number of CFU was determined by plate counting after 18- to 24 h incubation at 37°C. The ratio of the determined CFU number to the initial CFU number was defined as the recovery.

On the basis of the previous studies, the incubation time or the separation time was determined.

Table S1 Effect of amount of IMMPs on recovery （$\overline{x}$±*s*，*n=*5）

| Amount of IMMPs (µl) | Colony forming unit (CFU ml^-1^) | Recovery (%)^1^ |
| --- | --- | --- |
| 5 | 82.8±3.7 | 78.1±3.4 |
| 10 | 92.4±2.7 | 87.2±2.5 |
| 15 | 93.0±3.0 | 87.7±2.8 |
| 20 | 92.6±2.6 | 87.4±2.4 |
| 25 | 93.6±2.7 | 88.3±2.5 |
| 30 | 93.2±3.5 | 88.0±3.3 |

1. There were 106 CFU ml^-1^ in the sample.

Table S2 Effect of incubation time on recovery （$\overline{x}$±*s*，*n=*5）

| Incubation time (min) | Colony forming unit (CFU ml^-1^) | Recovery (%)^1^ |
| --- | --- | --- |
| 5 | 42.4±4.8 | 40.0±4.6 |
| 10 | 93.0±3.0 | 87.7±2.9 |
| 15 | 93.4±2.9 | 88.1±2.8 |
| 20 | 93.0±1.9 | 87.7±1.8 |
| 30 | 92.8±3.7 | 87.5±3.5 |

1. There were 106 CFU ml^-1^ in the sample.

Table. S3 Effect of separation on recovery （$\overline{x}$±*s*，*n=*5）

| Separation time (min) | Colony forming unit (CFU ml^-1^) | Recovery (%)^1^ |
| --- | --- | --- |
| 1 | 42.8±2.4 | 40.4±2.3 |
| 2 | 92.8±2.6 | 87.5±2.4 |
| 3 | 93.6±3.0 | 88.3±2.8 |
| 4 | 94.0±1.4 | 88.7±1.3 |
| 5 | 95.0±2.3 | 90.0±2.2 |

1. There were 106 CFU ml^-1^ in the sample.


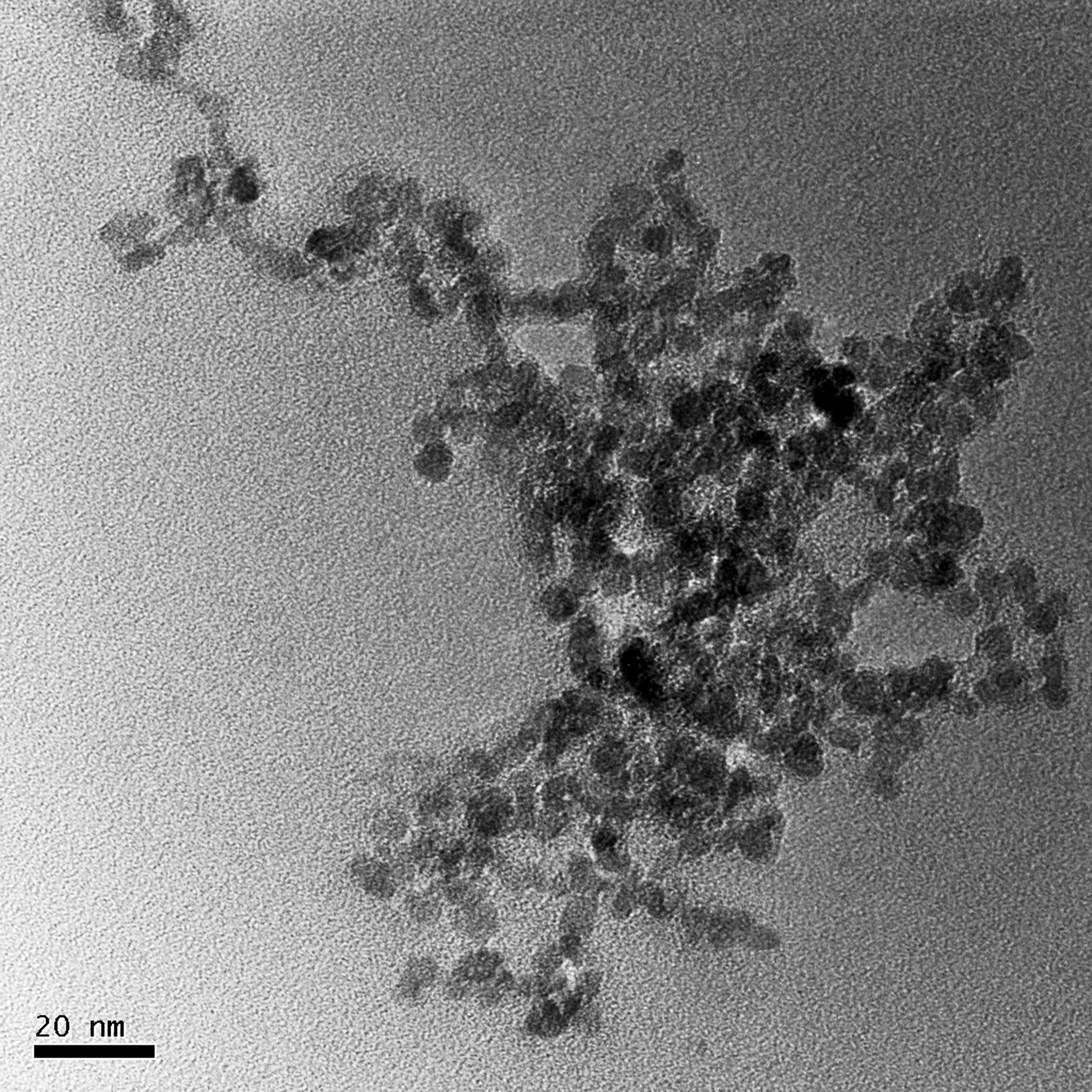


Fig.S1. Transmission electron micrograph of IMMPs. The electron dense core was about 5 nm.
